# Demonstrating a new technology for space debris removal using a bi-directional plasma thruster

**DOI:** 10.1038/s41598-018-32697-4

**Published:** 2018-09-26

**Authors:** Kazunori Takahashi, Christine Charles, Rod W. Boswell, Akira Ando

**Affiliations:** 1 Department of Electrical Engineering, Tohoku University, Sendai, 980–8579 Japan; 2 Space Plasma, Power and Propulsion Laboratory, Research School of Physics and Engineering, The Australian National University, Canberra ACT, 2601 Australia

## Abstract

Space debris removal from Earth orbit by using a satellite is an emergent technological challenge for sustainable human activities in space. In order to de-orbit debris it is necessary to impart a force to decelerate it, resulting in its atmospheric re-entry. A satellite using an energetic plasma beam directed at the debris will need to eject plasma in the opposite direction in a controlled manner in order to maintain a constant distance between it and the debris during the deorbiting mission. By employing a magnetic nozzle plasma thruster having two open source exits, bi-directional plasma ejection can be achieved using a single electric propulsion device. Both the forces exerted on the thruster and the target plate simulating the debris are simultaneously measured in a laboratory space simulation chamber showing that a force decelerating the debris and a zero net force on the thruster can be successfully obtained. These two forces can be individually controlled by external electrical parameters, resulting in the ability to switch the acceleration and deceleration modes of the satellite and the debris removal mode using a single electric propulsion device.

## Introduction

Space debris orbiting around the Earth has become a serious problem over the past few decades^1–3^; collisions with satellites not only cause damage to the spacecraft but can result in an increase in the amount of debris^4^. Indeed, investigations into the mass distribution have shown a continuously increasing mass of debris^5^. To preserve a secure space environment and reduce the risk of collisions, the active removal, or de-orbiting of space debris is an emergent technological challenge. One of the important targets of the active removal is large space debris in the low Earth orbit (LEO), typically weighing about a ton and being a few meters in size^6^.

If remedial action is not taken in the near future, it will be difficult to prevent the mass of debris increasing, and the production rate of new debris resulting from collisions will exceed the loss rate due to natural orbital decay^7^. Some concepts of space debris capture and removal have been proposed^8^; by using robotic arms and a tether net^9,10^, a laser-ablation-induced material ejection from the debris^11^, an orbit transfer using an electro-dynamic tether method^12^, and an ion-beam shepherd (IBS) method^13,14^ using two ion-beam sources.

**Figure 1:**
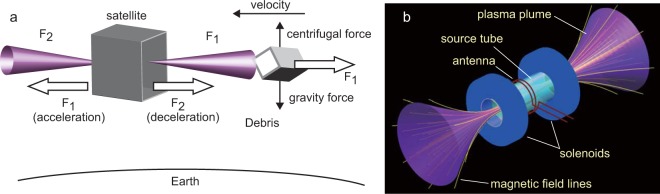
(**a**) Concept for space debris removal by bi-directional momentum ejection from a satellite. (**b**) Schematic of a magnetic nozzle rf plasma thruster having two open source exits. (**a**) When plasmas carrying momentum fluxes *F*_1_ and *F*_2_ are expelled from two axially opposite satellite exits, the respective forces shown by the horizontal arrows *F*_1_ (pointing to the left and providing the acceleration of the satellite with respect to the orbit velocity) and *F*_2_ (providing the deceleration) are generated and used to adjust the satellite velocity relative to the debris. Continuously imparting momentum flux *F*_1_ to the debris (horizontal arrow *F*_1_ pointing to the right) will cause its deceleration, final re-entry into the Earth atmosphere and natural burn up. (**b**) The open exits magnetic nozzle rf plasma thruster forming the single electric propulsion device where control of the momentum flux imparted onto the debris is obtained via the control of the plasma momentum fluxes ejected at each open exit using variable external parameters (solenoids currents and propellant gas flow rates).

Orbiting space debris has an angular momentum where the centrifugal force is balanced against the gravitational force and a constant altitude is maintained if no drag forces act on the debris (sketched in Fig. 1a). Most of the contactless concepts (laser-ablation and IBS) have proposed imparting a force to the debris thereby decelerating them in a direction opposite to their velocity to transfer them to a lower altitude where they finally re-enter the Earth’s atmosphere and naturally burn up. In the case of imparting a force to the debris by plasma ejection from a satellite using an electric propulsion device, such as the IBS method, the satellite is simultaneously propelled in the opposite direction, making it difficult to maintain the distance between the debris and the satellite. The IBS proposal would require two ion-gridded thrusters on the satellite as shown in Fig. 1a, one of which imparts a force to the debris and another balances the thrust by ejecting plasma in the direction opposite to the debris.

In general, the thrust (which is a force) is given by the rate of the change of momentum, corresponding to the flux of momentum ejected from the satellite, which can be derived from the momentum conservation law^15^. Considering the ejection of plasma having momentum flux *F*_1_ and *F*_2_ to the right and left sides of the satellite (Fig. 1a), the net thrust exerted on the satellite and the force on the debris are given as (*F*_2_ − *F*_1_) and *F*_1_, respectively, where the positive force is defined as the rightward direction. According to an analysis in ref.^13^, a deorbit time of between 80 and 150 days for a 1 to 2 tonne object would require a thruster performance of about 60 mN and a specific impulse of about 1800 sec. A similar analysis performed in ref.^16^ has also shown similar requirement of about 30 mN thrust at 2.5–3 kW power level and discussed the beam divergence effect on the momentum transfer efficiency to the debris, considering a safe distance to the debris to be about 7 m, where typical beam divergence for the ion gridded thrusters and the Hall thrusters are about 10–20 and 40–50 degrees half angles, respectively^17^. Therefore, the active removal of the large size debris clearly requires the high power electric propulsion device operated at the thrust level of several tens of mN at the power level of about a few kW.

Another key technology for debris removal by electric propulsion is the control of the acceleration and deceleration (defined as in Fig. 1a) of the satellite, which can be performed by adjusting the bi-directional plasma ejection. However, mounting two propulsion systems on the satellite will increase its development risk and make the satellite system integration more difficult due to weight and size considerations, which include the propulsion devices themselves, their power supplies and other necessary components (e.g., neutralizer, mass flow controller, etc). In such a propulsion system for the IBS mission, both the ion gridded thruster and the Hall thruster require two thrusters and two neutralizers so the actual specific impulse (effective utilization of the gas propellant) is lower than that evaluated for the single thruster.

For long-term operation of satellite for space debris removal, the lifetime of the propulsion device is one of the most important considerations. Operations of the ion gridded thrusters and the Hall thruster are known to be significantly affected by erosion of the electrodes and walls exposed to the plasma, especially for their high power operations. To overcome this problem, electrodeless plasma thrusters using magnetic nozzles (e.g., radiofrequency (rf) and helicon plasma thrusters^18,19^, a Variable Specific Impulse Magneto plasma Rocket^20^, and an electron cyclotron resonance thruster^21^) have been investigated, where the high density plasma produced inside the source is transported to the source exit along magnetic field lines. Various types of plasma acceleration processes can occur, e.g., ion acceleration by a current-free double layer^22^ and electromagnetic acceleration by an electron diamagnetic current^23^. Using a magnetic nozzle to increase thrust has recently been demonstrated by measurement of the force exerted on the magnetic field lines^24^. Experiments and simulations have shown that the thrust force is also affected by the neutral density profiles which affect the plasma density profile^25,26^. Measurement of ion energy distribution functions in a basic laboratory device has shown the spontaneous generation of supersonic ion beams on both the upstream and downstream sides of the plasma source^27^.

In the present study a bi-directional plasma ejection from a magnetic nozzle rf plasma thruster having two open source exits (Fig. 1b) is demonstrated in a laboratory experiment, where both the forces exerted on the thruster and a target plate simulating the debris are simultaneously measured. The measurement shows that by a judicious selection of experimental parameters, the force decelerating the debris can be imparted to the target while maintaining a zero net force on the thruster. This is accomplished by varying either the magnetic field configuration or propellant gas flow rates using two gas inlets located left and right of the source center. Consequently, the present rf plasma thruster can produce all three required operational modes in ONE electric propulsion device; the debris removal mode, the acceleration mode and the deceleration mode of the satellite, the latter two being useful for adjusting the satellite velocity relative to the debris.

The proposed thruster can be scaled up in size. The previously reported maximum value of the thrust imparted by the single open end thruster has been about 55–60 mN for about 6 kW rf power^28^, where the source diameter is 9.5 cm and larger than a 6.5-cm-diameter source tube used in the present paper. More recent experiments show a higher performance giving a thrust of 65–70 mN for about 6 kW rf power with a specific impulse of 2000–3000 sec in the laboratory test, where the location of the gas injection port is changed and the thruster efficiency is increased up to 20%^29^. We have found that a 30 mN thrust level can be obtained with rf power less than 1.5 kW and a specific impulse of 1500 sec. It should be mentioned that the thruster is not operated in Xe but rather Ar, which has a cost advantage. Furthermore, very efficient rf amplifiers and impedance tuning systems are also now under development^30–32^. Previously performed basic laboratory experiments have shown that the ion beam accelerated by a potential drop formed in a magnetic nozzle has a divergence half angle of at most about 20 degrees, even at the edge of the beam plume^33–35^, being very similar to that in the ion gridded thruster. Furthermore, results in this paper shows similar values of thrust for the acceleration/deceleration modes and force to the debris in the debris removal modes, implying the operation mode can be changed while maintaining the effective utilization of the gas propellant and the rf power. Based on these published results of research and development, the system in this paper can potentially satisfy the requirements for the IBS mission with a single thruster.

## Results

### Space debris removal experiment

**Figure 2:**
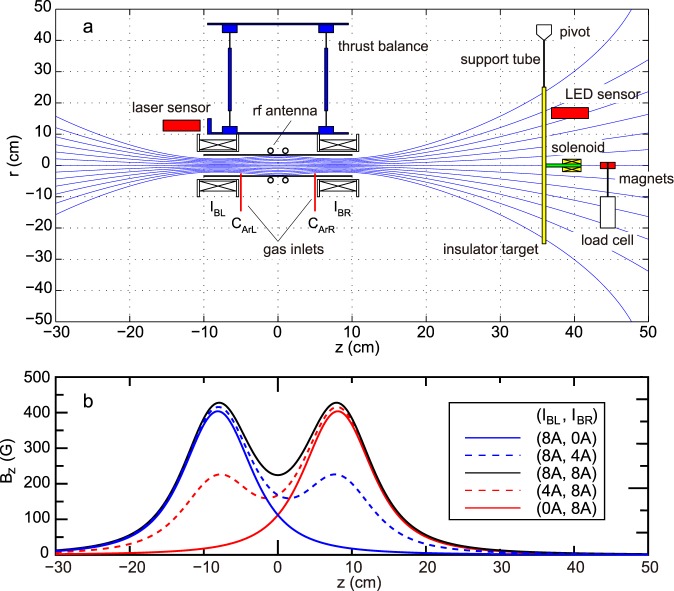
(**a**) Schematic diagram of the experimental setup, together with the calculated magnetic field lines for the (*I*_BL_*, I*_BR_) = (8 A, 8 A) Left/Right solenoidal current case. (**b**) Calculated magnetic field profiles on axis for various combinations of (*I*_BL_, *I*_BR_). Both the thruster (attached to the pendulum thrust balance) and separated insulating target acting as space debris are immersed in a space simulation chamber. The displacements of the thrust balance and the target plate are simultaneously measured and calibrated into forces (a positive value corresponds to a displacement and force pointing to the right).

The experiment is carried out with the magnetic nozzle rf plasma thruster having two open source exits on the left- and right-hand sides. The thruster comprises a 6.5-cm-inner-diameter 20-cm-long glass tube, two solenoids (axially centered at *z* = ±8.1 cm), and a rf antenna, the whole being attached to a pendulum thrust balance immersed in a 1 m diameter, 2 m long vacuum chamber. A schematic of the setup is shown in Fig. 2a. Argon gas is introduced from two gas inlets on the side wall of the source tube at *z* = ±5 cm with a total mass flow rate of 100 sccm, where the mass flow rates from the left and right inlets (*C*_ArL_ and *C*_ArR_) are individually controlled by two mass flow controllers. Two solenoids located near the two open source exits provide a magnetic field and the field configuration can be changed via the dc solenoid currents (*I*_BL_, *I*_BR_) as shown by the calculated magnetic field in Fig. 2b. A shielded double-turn rf loop antenna^36^ is situated at the axial center of the source tube (defined as *z* = 0) and powered from a 13.56 MHz, 1 kW rf generator; the power is primarily coupled to the electrons that ionise the argon gas by electron impact producing a high density plasma. Axial forces are imparted to the mechanical and magnetic structures of the thruster via momentum transfer by the ions interacting with the radial wall^37^ and by the Lorentz force on the magnetic field due to the plasma-induced electric current^23,24,38,39^. The thrust force exerted on the thruster by the plasma is obtained by measuring the displacement induced by the plasma ejection^19^. A 45-cm-diameter pendulum target plate simulating the debris is suspended from a pivot located at *z* = 35.9 cm downstream from and to the right of the thruster, and the force exerted on the target plate is obtained from its measured displacement combined with a calibration coefficient relating the displacement to the force^40^. The direction of both the forces and the displacements is defined as positive for the rightward direction with the detailed procedures for calibration of the two force measurements described in the Method section. An ion saturation current from an axially-movable and radially-facing Langmuir probe, which is proportional to a plasma density, is measured in addition to taking an image from a vacuum view port on the chamber side wall using a digital camera (where it should be mentioned that the target plate is not set in the chamber when taking the Langmuir probe data).

### Laboratory demonstration of the debris removal concept

**Figure 3:**
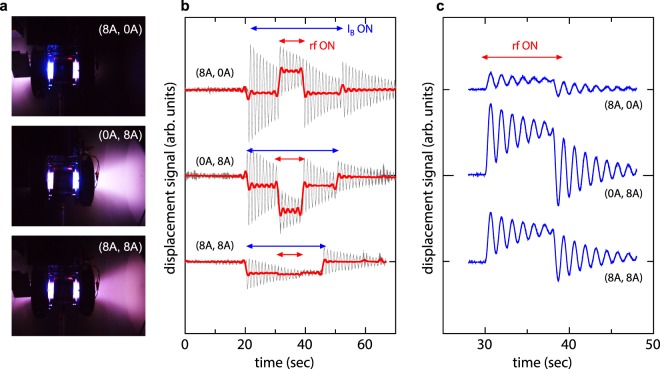
(**a**) Photographs taken by a digital camera via a vacuum viewport on the chamber side wall for the solenoid currents of (*I*_BL_, *I*_BR_) = (8 A, 0 A), (0 A, 8 A), and (8 A, 8 A). (**b**) The raw (gray thin lines) and filtered (red bold lines) displacement signals of the thruster attached to the thrust balance. (**c**) The displacement signals of the target plate. The positive and negative displacement in b and c corresponds to the rightward and leftward directions, respectively. (**a**) Shows that the plasma exhausted from the left- and right-hand open source exits are changed by the magnetic field configuration. The displacement signals in b show that the deceleration (rightward, *F*_2_ − *F*_1_ > 0) and acceleration (leftward, *F*_2_ − *F*_1_ < 0) forces are exerted to the thruster (satellite) for the (*I*_BL_, *I*_BR_) = (8 A, 0 A) and (0 A, 8 A) cases, respectively (Fig. 1a). The results for the (*I*_BL_, *I*_BR_) = (8 A, 8 A) case demonstrates that zero thrust force is exerted to the thruster (**b**) while imparting the force to the target (**c**).

Figure 3a shows photographs taken via a vacuum view port on the chamber side wall for solenoid currents of (*I*_BL_, *I*_BR_) = (8 A, 0 A), (0 A, 8 A), and (8 A, 8 A), with the gas flow rates from the two gas inlets respectively set at 50 sccm yielding a total flow rate of 100 sccm. A brighter plasma plume is seen on the left and right sides for the (*I*_BL_, *I*_BR_) = (8 A, 0 A) and (0 A, 8 A) cases, respectively, while bi-directional plasma plumes are observed for the symmetric magnetic field configuration of the (*I*_BL_, *I*_BR_) = (8 A, 8 A) case. The displacement signals of the thrust balance and the target plate for these conditions are shown in Fig. 3b,c, respectively. For the (*I*_BL_, *I*_BR_) = (8 A, 0 A) and (0 A, 8 A) cases shown at the top and middle of Fig. 3b,c, the thruster moves toward the right and the left respectively, demonstrating that changing the magnetic field configuration engenders the deceleration and acceleration modes respectively, while the target is always pushed towards the right. For equal solenoid currents, (*I*_BL_, *I*_BR_) = (8 A, 8 A), a symmetric magnetic field configuration is produced; the target plate moves to the right while a zero displacement of the thruster is maintained (bottom panels of Fig. 3b,c). These results show that this bi-directional rf plasma thruster can provide the three operation modes necessary for space debris removal (imparting the force to the debris with a simultaneous zero net force on the thruster).

In order to perform space debris removal in the space environment surrounding the Earth, the acceleration and deceleration of the thruster, the relative velocity with respect to the debris, and the force on the debris have to be precisely controlled by some external parameters. The control of the forces by the magnetic field configuration and the ratio of the propellant gas flow rates from the two inlets will be demonstrated in the next section.

### Control of the forces by the magnetic field configuration

**Figure 4:**
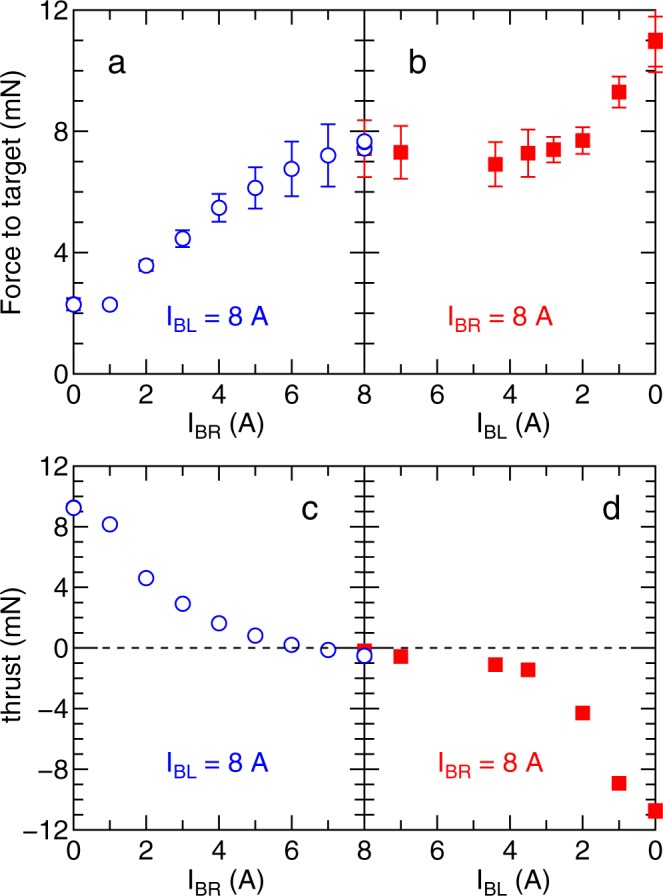
Simultaneously measured force to (**a**,**b**) the target and (**c**,**d**) the thruster as functions of the right- and left-hand solenoid currents (*I*_BR_ and *I*_BL_). Either of these two solenoid currents is maintained at 8 A when surveying the other one. The momentum flux ejection to the left- and right-hand sides of the thruster can be controlled by the magnetic field configuration, yielding the space debris removal mode (zero thrust and the finite force to the target), the acceleration mode (leftward force to the thruster), and the deceleration mode (rightward force to the thruster).

Figure 4 shows the measured forces on the target (Fig. 4a,b) and on the thruster (Fig. 4c,d) as a function of the currents supplied to the right and left solenoids (*I*_BR_ and *I*_BL_); when the current through one solenoid is changed, the current in the other solenoid is maintained at 8 A. It can be seen that for equal solenoid currents of 8 A (the symmetric magnetic field configuration), the thruster experiences a zero net force while a force of ~8mN is brought to bear on the target.

Interestingly, the force on the thruster can be changed, as seen in Fig. 4c,d, demonstrating that the bi-directional thruster is operational for both the modes of thruster acceleration (negative left directed force) and deceleration (positive right directed force). Between these three conditions, both the forces on the target and thruster continuously change when the solenoid currents are adjusted, resulting in a considerable degree of control of both forces. Additionally, the force to the target for (*I*_BL_, *I*_BR_) = (8 A, 8 A) case and the thrusts for (*I*_BL_, *I*_BR_) = (8 A, 0 A) and (0 A, 8 A) cases are very similar in the range of 8–10 mN; implying that the operation modes can be switched while maintaining both the effective utilization of the gas propellant and the rf power.

To understand the relation between the measured forces and the plasma behavior, measurement of the ion saturation current (proportional to the plasma density) is performed by two Langmuir probes mounted on an axially movable motor stage installed on the right side and on the fixed left side stage at *z* = −35.9 cm.

**Figure 5:**
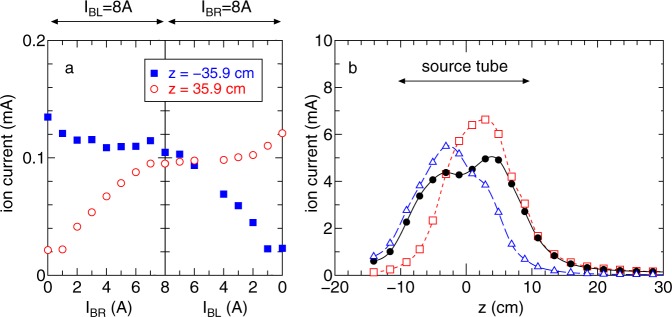
(**a**) Ion saturation currents measured by the Langmuir probes located at *z* = −35.9 cm (filled square) and +35.9 cm (open circles) as functions of the solenoid currents, where either of the two solenoid currents is maintained at 8 A. (**b**) Axial profiles of the ion saturation current for (*I*_BL_, *I*_BR_) = (8 A, 0 A) (open triangles), (8 A, 8 A) (filled circles), and (0 A, 8 A) (open squares), where the lines are added as visual guides. The plasma densities in the plumes exhausted to the left- and right-hand sides of the single propulsion device can be controlled by the magnetic field configuration, resulting from the change in the density profile inside the source tube. When the densities exhausted on both sides are balanced, the zero net thrust is maintained while imparting the force to the target. The solenoids are axially centered at *z* = ±8.1 cm (Fig. 2).

The ion saturation currents measured at *z* = ±35.9 cm are plotted in Fig. 5a as a function of the solenoid currents, showing that the density of the plasmas exhausted from the source to the left and right is continuously changed by the magnetic field configuration. In these cases, acceleration and deceleration forces on the thruster can be generated, while a zero net force to the thruster is maintained when the densities of the exhausted plasma to both sides are equal. Detailed axial measurements are performed for the three conditions of (*I*_BL_, *I*_BR_) = (8 A, 0 A), (8 A, 8 A), and (0 A, 8 A) as plotted in Fig. 5b. The maximum density region is found to be formed at the high magnetic field sides for the asymmetric field configuration, while a symmetric density profile is formed within the source tube for the equal solenoid currents case. Therefore, the symmetry of the axial density profile inside the source affects the plasma ejections to the left and right and consequently the forces exerted on the thruster and the target.

### Control of the forces by the gas flow rates

**Figure 6:**
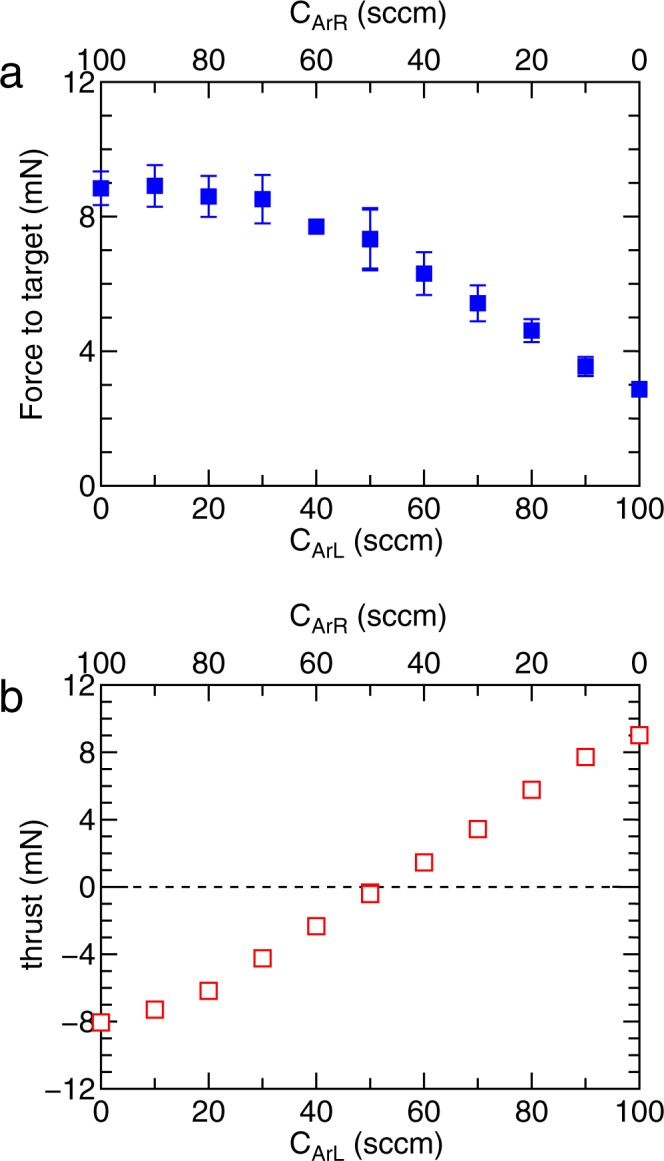
Simultaneously measured forces to (**a**) the target and (**b**) the thruster as functions of the gas flow rates from the left- and right-hand gas inlets (*C*_ArL_, *C*_ArR_), where the total gas flow rate is maintained at *C*_ArL_ + *C*_ArR_ = 100 sccm and the solenoid currents are set as (*I*_BL_, *I*_BR_) = (8 A, 8 A). The control of the momentum flux ejection to the left- and right-hand sides can also be obtained by changing the gas flow rates (using the inlets positioned at *z* = ±5 cm), yielding the space debris removal mode and the two acceleration/deceleration modes of the thruster/satellite.

**Figure 7:**
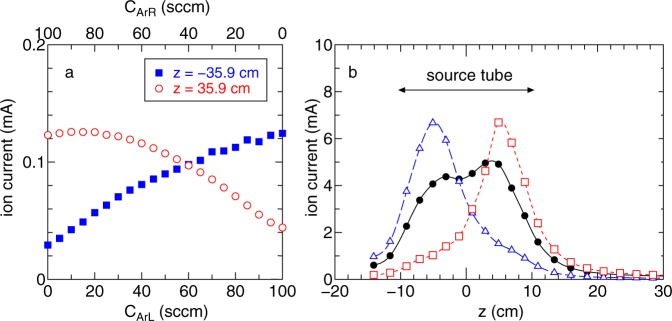
(**a**) Ion saturation currents measured by the Langmuir probes located at *z* = −35.9 cm (filled square) and +35.9 cm (open circles) as functions of the gas flow rates, where the total gas flow rate is maintained at *C*_ArL_ + *C*_ArR_ = 100 sccm and the solenoid currents are set as (*I*_BL_, *I*_BR_) = (8 A, 8 A). (**b**) Axial profiles of the ion saturation current for (*C*_ArL_, *C*_ArR_) = (100 sccm, 0 sccm) (open triangles), (50 sccm, 50 sccm) (filled circles), and (0 sccm, 100 sccm) (open squares), where the lines are added as visual guides. The densities exhausted from the source can be controlled by the gas flow rates by inducing changes in density profile inside the source tube. The maximum density appears very close to the gas inlets due to the high neutral density there. For equal gas flow rates from the two gas inlets, the plasma densities at the left- and right-hand sides are balanced; the zero net thrust and the force to the target is simultaneously obtained as shown in Fig. 6.

It should be noted that previous analytical models, experiments, and simulations have shown that the spatial profile of the neutral density significantly affects the plasma density profile^41^ and the thrust force^42,43^. Here the measurements of the forces are performed for various ratios of the gas flow rates from the two gas inlets placed at *z* = ±5 cm, as plotted in Fig. 6, where the solenoid currents are chosen as (*I*_BL_, *I*_BR_) = (8 A, 8 A) providing a symmetric magnetic field configuration and the total gas flow rate *C*_ArL_ + *C*_ArR_ is maintained at 100 sccm. Setting equal gas flow rates from both the left and right gas inlets as (*C*_ArL_, *C*_ArR_) = (50 sccm, 50 sccm) results in a zero net thrust force to the thruster and a right directed force to the target, while left and right directed forces to the thruster can be obtained by changing the ratio of the gas flow rates. The measured ion saturation currents at *z* = ±35.9 cm and the axial profiles as a function of the gas flow rates in Fig. 7 show a similar scenario to that discussed with Fig. 5. The asymmetry of the density profile in the source affects the densities of the plasma plumes exhausted to the left and right. The positions of the maximum density in Fig. 7 are close to the gas inlets which have a high neutral density and consequently a higher rate of the ionisation process.

## Conclusion

Laboratory experiments have shown that bi-directional plasma ejection from an open exits magnetic nozzle rf plasma thruster can impart a force to space debris whilst maintaining a zero net thrust force to the thruster (and satellite). The simultaneous measurements of the forces to the thruster and the target plate show that the two forces can be controlled by the magnetic field configuration and/or the gas flow rates from the left and right gas inlets respectively. Therefore, the system can be operational in three modes; the acceleration mode of the satellite, the deceleration mode of the satellite, and the space debris removal mode. These can be brought into play by simply switching electric signals to the solenoid current and the mass flow controller. As there is no movable mechanical structure this thruster configuration can provide new technology for the removal of space debris.

## Method

The thrust force and the force imparted to the target plate are obtained by multiplying the calibration coefficients to the measured displacements, following a method described previously in refs.^19,40^. Here application of this method to the present procedures for calibration of both forces is described:

### Thrust balance calibration

**Figure 8:**
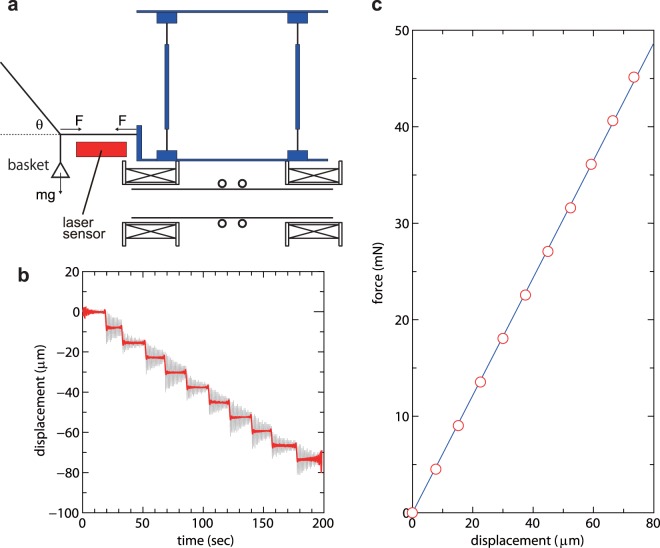
(**a**) Schematic diagram of the calibration basket and threads attached to the thrust balance. (**b**) Displacement signal of the laser sensor when putting a known mass to the basket at about every 20 sec (a gray line), together with a filtered signal (solid red line). (**c**) The relation between the applied horizontal force and the measured displacement (open circles), together with a fitted line (thin blue line) giving the calibration coefficient.

The coefficient relating the displacement to the force is obtained by measuring the displacement when applying a known force. A basket is tied to the back side of the thrust balance via a thin horizontal thread and a second thread attached to a support structure of the thrust stand as shown in Fig. 8(a). A small weight with a mass of about 0.323 g is put on the basket every 20 sec or so before pumping down the chamber; the displacement of the thruster structure is subsequently measured using the commercial laser displacement sensor as shown in Fig. 8(b), where the thin gray line is the signal obtained by the sensor. Since the signal includes the low frequency oscillation of the pendulum of frequency about 1 Hz, the frequency spectrum obtained via a Fourier transform is filtered using a low pass filter and further converted via an Inverse Fourier transform into the temporal signal as shown by the red line in Fig. 8(b). A force balance in static equilibrium gives the horizontal force as *F* = *mg* cot*θ*, where *m*, *g*, and *θ* are the total mass of the weight put on the basket, the gravitational acceleration, and the angle between the two threads. The relation between the applied horizontal force and the displacement is obtained as plotted by the open circles in Fig. 8(c) and the fitted solid line in Fig. 8(c) is obtained by a least squares method which gives the calibration coefficient.

### Target calibration

**Figure 9:**
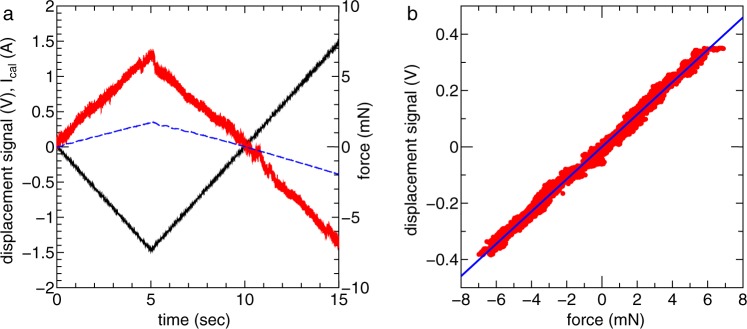
(**a**) Temporal evolution of the small solenoid current (*I*_cal_: solid black line), the force (solid red line) measured by the load cell, and the displacement signal from the LED sensor (dashed blue line). (**b**) The relation between the horizontal force imparted to the target structure and the displacement (red dots), together with a fitted line (solid blue line) giving the calibration coefficient.

The coefficient relating the target displacement to the force imparted to the target is obtained by the known-force-induced displacement as well as the thrust balance, where the displacement is measured by a commercial light emitting diode (LED) sensor. A small permanent magnet is located at the bottom wall of the chamber, being used as a magnetic damper to damp the oscillation of the pendulum motion. A small solenoid is attached to the target plate as shown in Fig. 2(a). The other permanent magnet mounted on a load cell, which is mechanically isolated from the target, is located behind the small solenoid. When supplying a dc solenoid current *I*_cal_ to the small solenoid, static and horizontal magnetic forces are exerted on both the permanent magnet and the solenoid, where the force to the permanent magnet is equal in magnitude and opposite in direction to that of the solenoid mounted on the target plate. Therefore, the magnitude of the magnetic force exerted to the target can be obtained from the signal of the load cell, which is input to a highly accurate differential amplifier with a gain of thousands and digitized by a 16 bit analog-digital convertor operated by a Labview program. The current *I*_cal_ is very slowly swept between ±1.5 A for about 10 sec as shown in Fig. 9(a); the displacement signal from the LED sensor and the absolute value of the horizontal force are simultaneously obtained as in Fig. 9(a). The data of the measured force and the displacement gives the relation plotted by the red dots in Fig. 9(b), shows good linearity between displacement and force and the fitting line shown as a solid blue line yields the calibration coefficient relating the displacement to the force to the target. The validity of the target technique has already been reported in ref.^40^ indicating a good agreement between the thrust values independently measured by the thrust balance and by the target method.
